# Supplementary material for: Mapping the evolution of fertility support policies in China: A content and instrumental analysis
Source: PLoS One. 2025 Oct 9;20(10):e0332137. doi: 10.1371/journal.pone.0332137 (PMC12510515; doi:10.1371/journal.pone.0332137)
Supplement: S1 Appendix — (ZIP) [file pone.0332137.s001.zip › S1 Appendix. 226 original policy documents/123-铁路女职工劳动保护实施细则(FBM-CLI-4-4944).doc]

铁路女职工劳动保护实施细则

**发布部门：** [铁道部（已撤销）](https://www.pkulaw.com/law/chl?Aggs.IssueDepartment=60215) [机构沿革](javascript:void(0))

**发布日期：**1991.01.11

**实施日期：**1991.03.01

**时效性：** [现行有效](https://www.pkulaw.com/law/chl?Aggs.TimelinessDic=01)

**效力级别：** [部门规章](https://www.pkulaw.com/law/chl?Aggs.EffectivenessDic=XE0301)

**法规类别：** [铁路运输](https://www.pkulaw.com/law/chl?Aggs.Category=06802) [劳动安全与劳动保护](https://www.pkulaw.com/law/chl?Aggs.Category=08706)

铁路女职工劳动保护实施细则
（１９９１年１月１１日铁道部发布）

　　**第一条**　为维护女职工的合法权益，切实搞好女职工劳动保护工作，根据国务院颁布的《[女职工劳动保护规定](https://www.pkulaw.com/chl/133408887a349cdabdfb.html)》，结合铁路实际，特制定本细则。

　　**第二条**　本细则适用于铁路各单位（包括全民和集体）女职工。

　　**第三条**　各单位必须严格执行国家和铁道部有关女职工劳动保护的法规，加强对女职工劳动保护的教育和培训。

　　**第四条**　凡适合妇女从事劳动的单位，不得拒绝招收女职工；适合女职工劳动的工种、岗位，应尽力安排女职工。

　　**第五条**　不得在女职工怀孕期、产期，哺乳期降低其基本工资，不得影响工资调整及有关福利待遇，不得因此提前解除劳动合同。

　　**第六条**　禁止安排女职工从事国家规定的第四级体力劳动强度的劳动以及其他女职工禁忌从事的劳动。

　　**第七条**　女职工在月经期间，所在单位不得安排其从事高空、低温和冷水、野外露天和国家规定的第三级体力劳动强度的劳动，月经期应尽可能调整其从事适合经期的轻工作；如不能调整轻工作时，根据工作和身体情况，给予经期假１－２天，不影响考勤。

　　**第八条**　女职工怀孕期间，所在单位不得安排从事国家规定的第三级体力劳动强度和孕妇禁忌从事的劳动，不得在正常劳动日以外延长劳动时间；对不能承受原劳动的，应根据医务部门证明，予以减轻劳动量或安排其他劳动。工程部门从事野外勘测工作及施工一线的女职工，应安排适当工作。

　　怀孕七个月以上（含七个月）的女职工，不得安排其从事夜班劳动；在劳动时间内应安排１－２小时休息时间。列车乘务员怀孕满五个月，应照顾下车安排适当工作。

　　怀孕的女职工，在劳动时间内进行产前检查，应当算作劳动时间。对生产一线的女职工，要相当地减少生产定额，以保证产前检查时间。

　　**第九条**　女职工产假（怀孕七个月以上）为９０天，其中产前１５天，难产增加１５天。多胞胎生育的，每多生育一个婴儿，增加产假１５天，属于计划生育政策的，按当地政府有关规定增加产假。教师正值寒、暑假期间生育，不顺延其休假时间。女职工在产假期间，不影响其原有福利待遇。女职工产假期满恢复工作后，应准许经过１－２周逐步恢复原劳动定额。

　　**第十条**　女职工怀孕不满四个月流产时，应根据医务部门意见，给予产假１５至３０天；怀孕４个月以上７个月以下流产时，给予产假４２天，以上产假期间工资照发。对有过两次以上自然流产史，现又无子女的女职工，应调离有可能直接或间接导致流产的作业岗位。

　　**第十一条**　有不满一周岁婴儿的女职工，需亲自哺乳的，其所在单位应当在每班劳动时间内给予其两次哺乳（含人工喂养）时间，每次３０分钟。多胞胎生育的，每多哺乳一个婴儿，每次哺乳时间增加３０分钟。女职工每班劳动时间内的两次哺乳时间，可以合并使用。哺乳时间和在本单位内哺乳往返途中的时间，算作劳动时间。

　　哺乳期满时，正值炎热季节（７－８月份），可根据当地气候条件延长１－２个月。对双胎或婴儿虽满周岁，因疾病、体弱经医务部门证明，哺乳时间可以适当延长，但最多不超过六个月。

　　**第十二条**　女职工在哺乳期内，所在单位不得安排其从事国家规定的第三级体力劳动强度的劳动和哺乳期禁忌从事的劳动，不得延长其劳动时间，一般不得安排其从事夜班劳动。女职工产假期满后，是否办理离岗休假，根据工作情况和自愿相结合的原则，由有关部门批准执行。在批准休假期间内，工资不得低于７５％。

　　**第十三条**　女职工在一百人以上的单位，应建立女职工卫生室，健全相应的制度并设专人管理，女职工在４０至１００人的单位和女职工占８０％以上的长途列车上，应设置简易的温水箱及冲洗器。流动分散工作的女职工，应发放单人自用外阴冲洗器。

　　女职工月经卫生费不应低于２元钱。

　　女职工比较集中的单位，应按照国家有关规定，建立孕妇休息室、哺乳室、托儿所、幼儿园等设施，要妥善解决女职工在生理卫生、哺乳、照料婴儿方面的困难。

　　**第十四条**　对女职工每两年进行一次妇科病普查，加强对妇科病预防、治疗工作，对已发生闭经、子宫脱垂、阴道滴虫等妇科疾病的女职工要加强管理，积极治疗，尽快恢复她们的健康。有条件的单位可设妇女保健门诊。

　　**第十五条**　经医务部门证明，患更年期综合症的女职工，不适合原岗位工作的，可改安排适当工作。

　　**第十六条**　女职工劳动保护的权益受到侵害时，有权向所在单位的主管部门或者当地劳动部门提出申诉，受理申诉的部门应当自收到申诉书之日起３０日内作出处理决定，女职工对处理决定不服的，可以在收到处理决定书之日赶快　，１５日内向人民法院起诉。

　　**第十七条**　对违反本细则侵害女职工劳动保护权益的单位负责人及其责任人员，其所在单位的主管部门，应当根据情节轻重，进行批评教育或给予行政处分，并责令该单位给予被侵害女职工合理的经济补偿；构成犯罪的，由司法机关依法追究刑事责任。

　　**第十八条**　各单位对本细则的执行情况，由劳动人事部门检查落实，工会、卫生、劳动保护部门对本细则的执行情况进行监督。

　　**第十九条**　女职工违反国家有关计划生育规定的，其劳动保护应按照国家有关计划生育规定办理，不适用本细则。

　　**第二十条**　本细则由中华全国铁路总工会负责解释。

　　**第二十一条**　本细则自一九九一年三月一日起实施。

　附：《铁路女职工劳动保护实施细则》中有关问题的说明：（略）

© 北大法宝：（[www.pkulaw.com](https://www.pkulaw.com/)) 专业提供法律信息、法学知识和法律软件领域各类解决方案。北大法宝为您提供丰富的参考资料，正式引用法规条文时请与标准文本核对。
欢迎查看所有[产品和服务](https://www.pkulaw.com/helps/2.html)。法宝快讯：[如何快速找到您需要的检索结果？](https://www.pkulaw.com/helps/70.html)[法宝](https://www.pkulaw.com/helps/69.html)V6有何新特色？

扫描二维码阅读原文

**原文链接：**<https://www.pkulaw.com/chl/262950e25c28007cbdfb.html>
